# Caregiver Employees’ Mental Well-Being in Hong Kong

**DOI:** 10.3390/healthcare12101013

**Published:** 2024-05-14

**Authors:** Maggie Man-Sin Lee, Eng-Kiong Yeoh, Kailu Wang, Eliza Lai-Yi Wong

**Affiliations:** The Jockey Club School of Public Health and Primary Care, The Chinese University of Hong Kong, Central Avenue, Hong Kong, China; 1155037369@link.cuhk.edu.hk (M.M.-S.L.); yeoh_ek@cuhk.edu.hk (E.-K.Y.); kailuwang@cuhk.edu.hk (K.W.)

**Keywords:** caregiver, aging, workplace, policies, spillover, role theory

## Abstract

Background: The rapidly aging global population has increased the demand for caregivers. Many caregivers simultaneously engage in paid employment, and the dual role makes the needs of caregiver employees conceivably more remarkable. However, there is a gap in the literature about the specific needs of caregiver employees. Method: Caregiver employees (n = 1205) across Hong Kong caring for those ≥65 years were recruited for a cross-sectional face-to-face survey from December 2021 to January 2022, to evaluate mental well-being measured by the Short Warwick –Edinburgh Mental Well-being Scale. Univariate and multivariate analyses were conducted; significant variables (*p* < 0.05) were included in multiple linear regression, along with caregiver-friendly workplace policies’ availability, to understand their association with their mental well-being. Findings: The mean score of the Short Warwick–Edinburgh Mental Well-being Scale among caregiver employees in this study was 24.9, with 7.2% indicative of probable clinical depression and 10.0% possible mild depression. In addition, the current study showed that 30.2% of the caregiver employees felt distressed about the caregiving role. Among external factors, family support (measured by the Lubben Social Network Scale) and workplace culture (measured by the Marshall Supervision Subscale) positively correlated with mental well-being with regression coefficients of 0.252 (*p* < 0.001) and 0.482 (*p* < 0.001), respectively. In the fully adjusted model, a negative regression coefficient was observed for overall spillover (−0.050, *p* < 0.001) and Short Warwick–Edinburgh Mental Well-being Scale scores, while positive regression coefficients were observed for overall self-rate (0.041, *p* < 0.001), Lubben (0.124, *p* < 0.001), and corporate culture (0.365, *p* < 0.001). Better Short Warwick–Edinburgh Mental Well-being Scale scores were observed when caregiver-friendly workplace policies were clearly stated than when they were made on a case-by-case discretionary basis. Conclusions: Caregiver-friendly workplace policies may be critical to Hong Kong’s sustainable future, both economically and socially, as they ensure a healthy and productive workforce to support an aging population.

## 1. Introduction

The rapidly aging global population has increased the demand for caregivers [1,2]. Family caregivers meet most caregiving needs, many simultaneously engaging in paid employment [1,3,4]. For instance, of the 43.5 million caregivers in the United States, 60% are caregiver employees (CE) [5]. Similarly, CEs comprise 67%, 75%, and 51.8% of the total caregiver population in the United Kingdom [6], Canada [7], and Japan [8], respectively.

In addition to the needs of the care recipient, there has been growing interest in the needs of caregivers [9,10], which may include psychological, informational, patient care, personal, spiritual, and household needs [11]. Unmet needs can significantly increase caregivers’ burden [12], strain, and the risk of poor mental health outcomes such as burnout, anxiety, and depression [13,14,15]. Although there is a gap in the literature about the specific needs of CEs, taking on an additional role as an employee makes the needs of CEs conceivably more remarkable. A national study in the US showed that most CEs are prone to being late, leaving early, taking time off, reducing work hours, or taking less demanding jobs [5].

Xiang et al. [16] suggest that the needs of CEs can be viewed from the perspective of disease progression of the care recipient, that is, at diagnosis, during treatment, terminal phase, and post-treatment survivorship. The high caregiver burden of CEs with sudden and unexpected changes relative to the care recipient’s condition could have a tremendously detrimental impact on their mental well-being, workplace productivity, and the net productivity of their employers.

Caregiver-friendly workplace policies (CFWPs) can reduce occupational and overall stress [17], moderate spillover effect (behaviors, emotions, and moods transferred between work and family roles) [15], promote health protective effects [7], and improve work motivations and productivity [18], thereby providing net economic benefits for both the CEs and the employers [19]. CFWPs could include appropriate workplace culture, targeted programs, and resources such as support services, paid leave, backup adult care, flexible work arrangements, and unpaid leave [7,20], providing greater flexibility and opportunities for CEs. For example, Fujihara et al. [21] showed that co-worker support lowers CEs’ burden, presenteeism, and overall work impairment among CEs for people with dementia.

Given the net positive impact of CFWPs, both governmental and private sectors are provisioning for such policies. For instance, in the United States, the Family and Medical Leave Act gives 12 weeks of unpaid leave over a 12-month period, albeit the eligibility criteria restricts this provision to private sector employers with at least 50 employees and requires that an employee work for at least 1250 h over the 12-month period [22]. However, 80% of the US employers surveyed provide CFWPs [23]. Similarly, Canada provides 28 weeks of Compassionate Care leave benefits with CFWP guidelines from the National Institute on Ageing [24,25]. Japan allows 93 days of caregiver leave per eligible family member [24].

Hong Kong ranks second in Asia after Japan in terms of population aging [26], with 18% of its population ≥65 years in 2019, which is projected to increase to 31% by 2044 [27]. Although the number and burden of caregivers in Hong Kong are set to rise, there are no reliable statistics on the overall caregiver population, let alone on the CEs, which is particularly concerning given that Hong Kong has a highly engaged workforce with 50.87% of its population engaging in paid employment [28]. In the one study we could identify in this domain, Ho Chan et al. [29] estimated that 7% of households had a caregiver in 2004. Moreover, earlier research from Hong Kong has explored gendered perspectives [30,31], age-based [32] and disease-specific [33,34,35] caregiving burdens, self-efficacy, or coping strategies, but not carers’ mental well-being or needs.

Therefore, this study aimed to assess the mental well-being of the CEs and the associated factors in Hong Kong to identify the needs and opportunities for future implementation of CFWPs. This study was guided by three research questions (RQs): (1) What is the mental well-being of the CEs? (2) Do CEs disclose their caregiver role in the workplace? (3) Which internal and external factors affect CE’s mental well-being?

## 2. Methodology

### 2.1. Ethics

The Chinese University of Hong Kong Survey and Behavioral Research Ethics Committee granted research ethics’ approval for this study (Reference No. SBRE(R)-21-011) on 8 December 2021.

### 2.2. Study Setting

Several contextual factors related to care recipients in Hong Kong make understanding CEs’ mental well-being imperative. Overall, one-third of the households studied have at least one elderly member [36], and only 2.3% of the elderly population is entitled to full subsidization [37]. In total, 65% of the elderly population has at least one, and one-third have ≥2 chronic conditions [38], which could require more extensive support [39]. While 64% of the households are nuclear families [36], the burdens shouldered by the CEs (key income earners) as the sole children of elderly parents are considerably intense. In addition, given that the population of children with special educational needs soared from 21,720 to 33,830 in the last half-decade [40], support for the “Sandwich generation” who are both caring for their young children and old parents becomes important [41]. In the cultural context, people of Hong Kong have generally inherited a strong sense of filial piety, demanding more from their supporting children [42]. Moreover, parental expectations have shifted from tangible support to emotional support [43], which could potentially challenge the mental health of the CE even further when self-efficacy and mental health are perpetual in nature.

Although some private companies provide generic support for general staff in Hong Kong, official caregiver support has not yet been observed [44]. The situation is particularly challenging when CEs’ protection is not discussed in law, public policy, or within organizations. Therefore, it is urgent to assess the needs of the CEs and explore the possibility of implementing CFWPs in Hong Kong’s specific context.

### 2.3. Study Design and Participants

CEs in Hong Kong were recruited for a cross-sectional face-to-face survey from December 2021 to January 2022, to evaluate mental well-being measured by the Short Warwick–Edinburgh Mental Well-being Scale (SWEMWBS). The inclusion criteria comprised any type of paid employment (full-time employment, part-time employment, or both), providing care to at least one recipient ≥65 years of age with a self-defined chronic condition, literate in English or Chinese, and able to provide consent to participate in the study. Verbal consent was obtained from all survey respondents, and participants were assured of their rights and freedom to withdraw from the study at any time. The information and responses of the participants were treated confidentially. All project data were anonymized and kept in password-protected folders only accessible to the lead author and the study supervisors.

Participants were recruited through the convenient quota sampling technique across the sixteen major industries in Hong Kong based on Census Quarter One employment distribution [45]. The sampling of participants according to the industry quota is described in detail in Supplementary Table S1. Street-level data collection was conducted by trained investigators from MOV Data Collection Limited throughout 18 districts of Hong Kong.

The sample size estimation was based on a mean SWEMWBS score of 21.78 with a standard deviation of 5.44 based on the study by McMahon and Cassidy [46] that recruited 326 family caregivers for a person with dementia, and the calculation method (estimation of confidence interval of one mean) was as described by Meeker et al. [47]. Given a standard deviation of 5.44 of target measurement and a two-sided confidence level of 95%, a sample size of 1000 produced a confidence interval with a width of ±0.337. The target sample size was increased by 100 to 1100 in a buffer for invalid responses.

### 2.4. Theoretical Framework

This study relies on three theories (spillover theory, Lazarus and Folkman’s stress and coping theory, and intersectionality) to explain the CE’s needs and behaviors. Spillover theory postulates the crossover impact between the work–family microsystems [48]. Spillover regarding time, energy, and behavior is generally negative if work–family interactions are rigidly constructed [48]. Stephens et al. [49] reported that stress in both employment and caregiving roles is significantly related to negative spillover, and negative spillover between the two roles is significantly related to depression.

Lazarus and Folkman’s stress and coping theory (for brevity, hereafter referred to as Stress Theory) postulates stress as a relational concept between individuals and their environment [50]. The stress model was first developed by Pearlin et al. [51] to examine the predictors of the caregiving burden, which has been positively associated with old age [52] and the female gender [53,54], and negatively with the size of the social support network [55,56,57].

Intersectionality is an analytical framework attempting to explain how the co-occurrence of disadvantaged identities can impact the individuals involved [58]. This framework has been frequently used to explain the difficulties experienced by caregivers when a caregiving role intersects with other identity factors like gender [59] and race [60].

**Figure 1:**
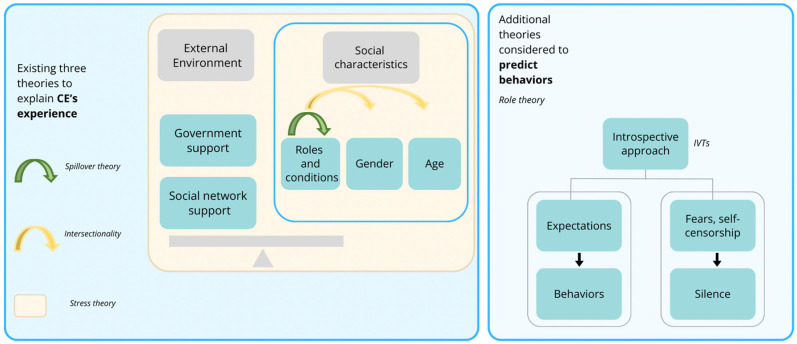
Illustrative diagram of the theoretical framework [48,49,51,52,53,54,55,56,57,58,59,60,61,62,63].

These three theories have a commonality in their structure, which is the consideration of the CE’s social characteristics (i.e., roles and conditions of caregivers and employment, gender, and age) and/or external factors (i.e., environment) laterally to understand the experience of the CEs through the interactivity of the variable factors (Figure 1).

### 2.5. Survey Instrument

The study questionnaire was developed based on a secondary data analysis of our previous qualitative study [44], in which in-depth semi-structured face-to-face interviews were conducted with nine CEs, three healthcare professionals, and three company management personnel about CFWPs in the context of Hong Kong. Our results indicated that the spillover effects and implicit voice theories (IVTs) were applicable in the context of Hong Kong [61,62]. The spillover effect can be positive and negative depending on the circumstances, but the “inseparable” nature of the dual roles contributes to the spillover effects. The mechanism of the spillover effect is conceptualized as one of the findings of “role struggle” underlined by role theory, which postulates interactions between individuals in organizations by focusing on their roles—that individual expectations underlie how he or she should perform appropriately in the roles [63]. Such role expectations for CEs, as caregivers and workers, influence their behavior and experiences at work and home, leading to spillover effects. IVTs can be applied to CEs to explain their silence at work regarding their caregiving role for fear of censorship or criticism.

In addition, our case study identified three dimensions affecting the experience of CEs: (1) external factors (workplace culture, social welfare, healthcare, and family and friends support), (2) internal factors (role balancing with the use of spillover theory and their perception of being in the role of CE in a corporate hierarchy), and (3) the sociodemographic background of the CEs and care recipients (Supplementary Table S2). Guided by these findings, a questionnaire was developed for the current study comprising five parts as follows:

Part 1 of the questionnaire (12 items) included questions related to screening for eligibility criteria, caregiving history, and average caregiving frequency.

Part 2 involved sociodemographic characteristics of the care recipients (12 items) and CEs (14 items).

Part 3 comprised seven SWEMWBS questions about mental well-being rated on a 5-point Likert scale [64]. An additional item was included to assess caregiving burnout [65]. SWEMWBS has been previously translated into Traditional Chinese and validated in the context of Hong Kong [64]. The SWEMWBS has also been tested in the Chinese population for validity, internal consistency, and psychometric properties [66], and for the measurement of the mental well-being of caregivers [67,68]. Permission was obtained to use the SWEMWBS for this study.

Part 4 included 50 framework questions based on three theoretical frameworks and our previous case study. Four validated tools were incorporated in this part: (1) Marshall Supervision Subscale of the Job Role Quality Questionnaire (external factors) [69]; (2) LEAP Leadership Behaviors and Organizational Climate Survey under work culture (external factors) [70]; (3) Lubben Social Network Scale (LSNS-6) under family and friend support (external factors) [71]; and (4) Inter-role conflict scales under the spillover effect (internal factors) [72]. While questions about social welfare and healthcare support availability had binary responses, subjective perceptions/experiences were measured on a 4-point Likert scale (1—most negative and 4—most positive).

Part 5 included four questions about the currently available CFWPs, a preference for the specific type of CFWP implementation in future policy recommendations, and a discussion of whether a suitable company policy can help CEs balance the roles of employees and caregivers more effectively. For the preference for the specific type of CFWP implementation in the future (questionnaire item E2), participants were asked to rate the importance of specific CFWPs on a scale of 1 (least important) to 10 (most important).

The complete questionnaire is presented in the Supplementary Materials.

### 2.6. Pilot Test

Before launching the primary survey, 10 participants (Supplementary Table S3) were invited to test the survey questionnaire’s comprehensiveness, relevance, understandability, and feasibility. The following questions were asked at the end of the interview to refine the questionnaire: (1) Do you think the questions covered all your concerns? (2) Any more questions to be added? The results indicate that the questionnaire covered all concerns and fit the local context, so no questions were deleted. However, sentences were rephrased with elaboration according to local wording and understanding. The response rate in the pilot test was 100%, with no rejection or missing data. Each questionnaire was completed in 20–40 min.

### 2.7. Statistical Analysis

CE characteristics were expressed as means ± standard deviations (SD), median (interquartile range; IQR), and frequency (%) as appropriate. Univariate and multivariate analyses were conducted (Mann–Whitney U Test and Kruskal–Wallis H Test by ranks for categorical variables and Spearman’s correlations for continuous variables) between independent and dependent variables (SWEMWBS score). Independent variables included (1) sociodemographic of the CE, (2) external and (3) internal factors, and (4) policy availability that would impact mental well-being (Supplementary Table S4). Controlled variables included demographic and health information of the care recipients. Cronbach’s alpha was used to test the internal consistency and reliability of the scores of individual instruments [73]. Cronbach’s alpha above 0.7 was considered good, and ≥0.8 very good [74].

Statistically significant variables (*p*-values < 0.05) were then included in the following regression analyses. Multiple linear regression was adopted to analyze how the internal/external factors and policy availability are associated with the SWEMWBS score [75]. Policy availability was recorded for each policy option clearly stated with code 0, option available at discretion with code 1, option not at all, and unknown with code 2 for regression. The original 16 categories of industries in which the CE was involved were regrouped into 8 categories based on census grouping for the regression analysis (See Supplementary Table S1). Since Kolmogorov–Smirnov [0.136 (*p* < 0.001)] and Shapiro–Wilk [0.962 (*p* < 0.001)] tests indicated that the SWEMWBS scores were not normally distributed (Supplementary Figure S1), a linear stepwise backward regression was used.

The regressions were conducted using two models. Model 1 (M1) considered the internal and external factors by subscales (dimensional), while Model 2 (M2) considered them in their entirety (summative). The complete results of the regressions are available in Supplementary Tables S5 and S6. Histograms of the residuals for Models 1 and 2 are presented in Supplementary Figures S2 and S3, which are both normally distributed. Multicollinearity was tested prior to multiple regressions using the variance inflation factor (VIF), and the variables with VIFs > 5 were excluded (Supplementary Table S7).

STATA statistical software version 15 (StataCorp LLC, College Station, TX, USA) and Statistical Package for Social Sciences (SPSS) Software (Version 17.0, SPSS, Inc., Chicago, IL, USA) were used for the analysis.

## 3. Results

The detailed sociodemographic characteristics of the interviewed CEs (n = 1205) are presented in Table 1. Briefly, 55.2% of the participants were females, over half of the respondents were in the 40–49 (27.4%) and 50–59 (23.9%) age groups, 69.8% had tertiary education, 78.6% held full-time employment, and 41.7% provided three to five hours of care per day. The median caregiving experience of the participants was ten years. The most common caregiving dimensions were decision-making involvement (81.1%) and financial support (80.7%). The proportion of respondents who reported household income above HKD 50,000 was 22.9%, while 22.8% reported HKD 30,000–39,999.

### 3.1. CEs’ Mental Well-Being

The median SWEMWBS score of our study population was 24 (IQR 21–28) (Table 2). According to the Warwick Medical School [77] cutoff, 7.2% of the CEs indicated signs of probable clinical depression, and another 10.0% indicated signs of possible mild depression. Within the sex dimensions of SWEMWBS scores, the highest mean score was feeling useful (3.7), with a standard deviation of 0.9 (Table 2).

### 3.2. Univariate Analysis between Demographic Factors and SWEMWBS

Educational attainment (*p* = 0.001), average caregiving hours per day (*p* = 0.001), household income (*p* < 0.001), employing industry (*p* < 0.001), work mode (*p* = 0.019), disclosure of CE status (*p* < 0.001), and care recipient’s utilization of public healthcare services (*p* < 0.001) were significantly associated with mean SWEMWBS scores, with the lowest scores observed for those who provided three to five hours of care per day, earned less than HKD 25,000, engaged in the accommodation and food services, had a part-time work status, did not disclose CE status, and non-utilization of public healthcare services (Table 3).

Spearman’s correlation was used for continuous independent variables, such as the age of the CE and the care recipient, total caregiving experiences, CE’s involvement in caregiving dimensions, care recipient’s number of social welfare items, number of comorbidities, and number of supports received in caregiving dimensions (Table 4). Total caregiving experience (ρ = 0.088, *p* = 0.002) and care recipient’s number of comorbidities (ρ = 0.091, *p* = 0.002) positively correlated with SWEMWBS scores. Care recipients’ number of social welfare items negatively correlated with SWEMWBS scores (ρ = −0.115; *p* < 0.001). The more the care recipients received in caregiving dimensions support, the better the SWEMWBS score: for physical care at the co-efficient 0.192 (*p* < 0.001); emotional care at 0.282 (*p* < 0.001); financial support at 0.185 (*p* < 0.001); and decision-making involvement at 0.145 (*p* < 0.001).

### 3.3. Reliability Analysis of Scores of Internal and External Factors and SWEMWBS

The Cronbach’s alpha coefficient of reliability for the scores of inter-role conflict (questionnaire items: D28a, D28b, D28c, D28d, D28e, D29a, D29b, D29c, D29d, and D29e), self-rating (questionnaire items: D32a and D32b), Lubben Social Network (questionnaire items: D21a, D21b, D21c, D22a, D22b, and D22c), the composite of the Marshall Supervision Subscale of the Job Role Quality (questionnaire items: D2-reversed for analysis, D3a, and D3b), and the LEAP Leadership Behaviors and Organizational Climate scales for corporate culture, was 0.958, 0.755, 0.916, and 0.814, respectively.

The composite score of self-rating was composed of only two items, which explained the relatively low but still very acceptable Cronbach’s alpha of 0.755. Cronbach’s alpha of 0.935 (seven items) for mental well-being indicated very good internal consistency of score data, and item redundancy was unlikely.

### 3.4. Univariate Analysis between the Internal and External Factors and SWEMWBS Scores

After ensuring the internal consistency of the internal and external factors and well-being measurements, a univariate analysis was conducted to map out the direct association. Spearman’s correlation showed significant associations between the internal/external factors and the well-being measurements, all with *p* < 0.001 (Table 5). Except for the spillover effect, which negatively correlated with SWEMWBS, positive correlations were observed with all other factors.

### 3.5. Univariate Regression Analysis between Policy Availability and SWEMWBS Scores

Univariate regression analyses were performed between the current policy availability and the well-being measurements (Table 6). Caregiver-inclusive corporate culture (*p* < 0.001), paid caregiver leave (*p* < 0.001), unpaid caregiver leave (*p* < 0.001), flexible working hours (*p* < 0.001), flexible work locations (*p* < 0.001), switch to part-time mode (*p* < 0.001), unpaid leave (*p* < 0.001), medical needs aid/insurance for employees’ parents (*p* < 0.001), and caregiving information, carer skills and guide to community care resources (*p* < 0.001) were significantly associated with mean SWEMWBS scores. SWEMWBS scores were lower when CFWPs related to caregiver-inclusive corporate culture, paid caregiver leave, unpaid caregiver leave, flexible working hours, flexible work locations, and medical needs aid/insurance for employees’ parents were discretionary versus if clearly stated. However, for CFWPs related to the switch to part-time mode, unpaid leave, caregiving information, carer skills, and guide to community care resources, higher SWEMWBS scores were noted if the policy was discretionary. There was no association between SWEMWSB scores and bereavement leave.

### 3.6. Multivariate Regression Analysis for the SWEMWBS Scores

Table 7 shows the regression results for internal and external factors for both Model 1 and Model 2 concerning SWEMWBS. Positive regression coefficients were observed for self-rating for family (0.039, *p* < 0.001) and work roles (0.044, *p* < 0.001), family support (0.252, *p* < 0.001), and corporate culture according to the Marshall Supervision Subscale (0.482, *p* < 0.001) in Model 1. In Model 2, a negative regression coefficient was observed for overall spillover (−0.050, *p* < 0.001), while positive regression coefficients were observed for overall self-rate (0.041, *p* < 0.001), Lubben (0.124, *p* < 0.001), and corporate culture (0.365, *p* < 0.001). Since this study aims to assess the effect of specific predictors or explanatory variables (namely, spillover effects, self-rating, family and friends support, and corporate culture) on the dependent variable (i.e., mental well-being), adjusted R2 values of 0.4038 and 0.3979 are considered acceptable on the condition that some of the variables are statistically significant [78].

We considered Model 1 for understanding the relationship between policy availability and mental well-being since the subscale analysis rendered a more precise view of the covariate’s correlation with the well-being measurements (Supplementary Table S5). For SWEMWBS scores, the care recipient’s items of social welfare had a negative coefficient of −1.117 (*p* < 0.001). At the same time, there was a positive correlation between the number of comorbidities of the care recipient at 0.308 (*p* = 0.015) and the support of decision-making participation received by the care recipient at 0.476 (*p* = 0.009). Those with a household income of HKD 50,000 or more were better off than those who earned less than HKD 25,000 by 1.115 (*p* = 0.012). Regarding the availability of policies, caregiver-inclusive corporate culture (variable name “E1a”) and flexible work locations (variable name “E1f”) had a positive impact on mental well-being when they were clearly stated, rather than discretionary, by 1.050 (*p* = 0.027) and 0.941 (*p* = 0.038), respectively.

Model 2 showed that all internal and external total scores significantly correlated with SWEMWBS scores, all with *p*-values < 0.001 (spillover at −0.050, self-rating at 0.041, Lubben at 0.124, and corporate culture at 0.356).

### 3.7. CE Status Disclosure

Around 76% of the survey respondents chose not to disclose their CE status. The most common reason for non-disclosure was that they seldom shared private matters at work (n = 476, 52.8%), followed by “Not necessary to disclose because I need no help” (n = 309, 34.3%), “Not necessary to disclose because I can handle it well” (n = 227, 25.2%), “Not necessary to disclose because the unsympathetic environment” (n = 175, 19.4%), “Afraid of being misunderstood” (n = 55, 6.1%), and “Afraid of unfair treatment” (n = 38, 4.2%).

### 3.8. CE’s Sense of Distress, Services Adoption, and Policy Preference

In total, 30.1% of the survey respondents reported distress while delivering caregiving duties. Additionally, 12.2% had used home care/homemaking/meals services/home volunteer services, 10.8% used home-based nursing services, 89.1% used either public or private healthcare services, and 20.2% used allied health services, including physical therapy services, occupational therapy services, and speech therapy services.

In terms of the perceived importance of specific CFWPs, participants most highly rated bereavement leave (mean ± SD: 8.3 ± 0.05) followed by flexible working hours (8.0 ± 0.05), aiding medical needs/insurance of employees’ parents (7.9 ± 0.05), caregiver-inclusive corporate culture (7.9 ± 0.05), paid caregiver leave (not counting paternity/maternity leave) (7.7 ± 0.05), flexible work locations (7.7 ± 0.05), unpaid caregiver leave (7.6 ± 0.05), information/carer skills/guide to community care resources (7.4 ± 0.05), switch to a part-time mode (7.3 ± 0.05), and unpaid leave (7.3 ± 0.05).

## 4. Discussion

The mean score of the SWEMWBS among CEs in this study was 24.91, indicative of better mental well-being, for instance, compared to 23.5 in the UK’s national general population (aged 16 and above) [79]. Overall, caregiving experience positively correlated with CE’s mental well-being. Nevertheless, 7.2% of CEs in this study demonstrated probable clinical depression and 9.96% possible mild depression, which is lower than that reported among Hong Kong’s CEs for Alzheimer’s disease patients (46.2%) [80]. Around one-third of the Alzheimer’s study sample gave care for more than 20 h per week [80], while 59% of the participants in the current study provided more than 20 h of care per week. The difference could be attributed to the type of care provided by the two groups: CEs in the current studies focus on decision-making and financial support, while Alzheimer’s patients require relatively more physical care [81], which could be a more significant risk factor for depression.

Care recipients’ support in decision making was more likely to contribute to the mental state of CEs than physical care, emotional care, or financial support. Fittingly, the most common dimensions of care provided by CEs were involvement in decision making, followed by financial support, physical care, and emotional care. Although the exact mechanism of the association between CE’s involvement in decision making and better mental health outcomes is unclear, involving themselves in decision making likely makes CEs feel part of the team as they can exchange opinions and share responsibility when deciding what is best for the care recipients. 

Similarly, a lower proportion of CEs in this study reported feeling distressed about the caregiving role than general caregivers for dementia patients in Hong Kong (30.2% vs. 40.2%) [65]. Although the same set of questions on service utilization had been asked, care recipients under the care of the CEs had used more formal care services than the general caregivers, 12.2% in home care services compared to 1.7% of the general caregivers, 10.8% in home-based nursing services compared to 6.1%, and 20.2% in allied health services compared to 4.2% [65]. The higher use of services could contribute to lower levels of burnout among CEs.

Further, CE’s mental well-being was positively related to the number of comorbidities of the care recipients and negatively related to the number of social welfare items. The association with the number of comorbidities could have arisen because the absolute sum of the items did not consider the severity, stage, or type of comorbidities. It is suggested that intersectionality between patient and caregiver factors, e.g., shift workers caring for end-stage cancer patients, should be examined to clarify which CEs are at increased risk for poor outcomes [16]. In the current context of Hong Kong, a disease-oriented study intersecting with the CE factor is essential for formulating future policies to prioritize resource allocation. For the association with the number of social welfare items used, the more social services the care recipients use might imply higher functional and emotional needs. We postulate that the care recipients might need more support from the CEs, negatively impacting CEs’ mental well-being.

Internal and external factors were highly predictive of CEs’ mental well-being. The spillover effect showed how work–family conflict negatively affected CEs’ mental well-being. It is consistent with the earlier study that there are negative spillover effects for employed adult caregiver daughters who must care for elderly parents while engaging in paid employment [49]. Others have shown that negative spillover conflict is positively associated with caregiving responsibilities and can lead to time theft [82,83].

Earlier studies have indicated that supportive managers, bank of trust [84], role balancing [44,61], and role empowerment by preparation and education [44] may act as enablers for CEs to balance the dual roles and live up to their duties effectively and efficiently. Consistent with these observations, the Marshall Supervision Subscale was positively related to the SWEMWBS scores, indicating the importance of corporate culture for CEs to balance the dual roles and improve CEs’ mental well-being. Moreover, the better CEs rated their performance in the roles (as a family and as an employee), the better their mental well-being. The relationship between self-rating and SWEMWBS scores is probably not causal but predictive. Family and friend support, as measured by the Lubben Social Network Scale, was also positively related to CEs’ mental well-being. This is consistent with a previous study from Hong Kong, which showed that social support acts as a moderator of stress coping in depression among older people facing stressful life events [85].

Regarding the availability of CFWPs and mental well-being, explicit caregiver-inclusive corporate culture had a more positive effect on mental well-being than discretionary culture. This is consistent with the previous finding that corporate culture, as one of the external factors, is of great importance to CEs’ mental well-being [44]. Clearly stating flexible work location arrangements rather than a discretionary decision on a case-by-case basis is also crucial for the mental well-being of CEs. It could be because, during COVID-19, flexible work location arrangements became more common, so uncertainty about the policy could cause confusion and worry.

Furthermore, most participants (74.4%) in this study had not disclosed their CE status, primarily due to an unsympathetic environment. This finding indicated that CEs in Hong Kong perceive risks in disclosing their CE status in the company hierarchy. Discrimination and stigmatization [44,84], apathy [44], fears, and self-scrutiny/judgment [44,61,62] are barriers to achieving a healthy work–life balance. Moreover, in the local context of Hong Kong, the prevalent concept of “work–life separation”—that personal matters should not be imposed on the workplace—hinders a healthy work–life balance [86]. While more flexible and permeable boundaries are associated with work–life enhancement [87], Hong Kong’s prevailing corporate culture generally appears quite the opposite.

The situation is further compounded by a system not geared to support CEs. Family caregivers are rarely identified in the care recipients’ health records [44]. Support for carers is available at community centers open from 9 am to 5 pm, but accessing such services may not be feasible for CEs [44]. Inadequate social welfare support or difficulties accessing healthcare support can significantly hinder CEs’ well-being [44]. Amidst the overwork culture [88], employees in Hong Kong are left with little space to process the possible fears or prejudices they face in coping with their new status as a CE.

Our findings strongly suggest that CFWPs can directly improve the mental well-being and organizational performance of CEs in Hong Kong. When improving mental well-being leads to a direct improvement in productivity [18,89], the benefits of CFWPs may be more direct than ever anticipated. Not only does this remove the stigma that a CE is only associated with productivity losses and workflow disruptions, but these studies also open opportunities for companies to invest in CFWPs.

Thus, our findings warrant exploring policies where CEs feel welcomed at their workplace and comfortable disclosing their status. In addition, governmental, healthcare, community, and private stakeholders should consider implementing support systems to relieve some of the burden. For instance, a meta-analytic study by Sörensen et al. [90] demonstrated that psychoeducational interventions such as verbal or written information about the care recipient’s likely clinical course or caregiver-specific resources, services, and training; supportive interventions like support groups, availability of care professions to give the primary career some time off; and psychotherapy are effective in reducing caregiver’s perceived burden and depression. More recently, e-health modalities, which may be particularly attractive considering the time constraints of CEs, have been shown to improve caregivers’ depression and quality of life but not their burden [91]. Moreover, the positive effects of e-health interventions may not last beyond the duration of the treatment [92]. During the COVID-19 pandemic, educational workplace interventions to increase awareness and organizational support to CEs have been shown to favorably impact CEs’ morale, job satisfaction, depression, psychosocial status, and self-reported health, with potential economic benefits for employers [93,94]. However, such interventions are yet to be tested in the context of Hong Kong and given the broad implications on the health of the working-age carers, older care recipients, and incentive for business to improve employee productivity and welfare, future research should actively investigate and identify effective interventions for CEs in Hong Kong.

## 5. Limitations

All data were collected on the street for 20–45 min, which could result in a fatigue response. In addition, we hypothesized the spillover effect between dual roles for CEs without a control group with only a single role. Furthermore, the survey was conducted as Hong Kong was recovering from the impact of the COVID-19 pandemic [95]. The well-being of the CEs could be heavily impacted due to city-wide restrictions to social life and mobility, emergency measures of the hospitals and the ancillary community health services, poor economic prospects, and fears of COVID-19.

## 6. Conclusions

CFWPs may be critical to Hong Kong’s sustainable future, both economically and socially, as it ensures a healthy and productive workforce to support an aging population. Institutional changes are essential, such as providing caregiver support services at worker-friendly hours, registering informal caregivers in the trust system for stakeholders’ use, and promoting awareness of CEs in the workplace. Governmental, healthcare, community, and private stakeholders can all play important roles in implementing CFWPs. Future research on CFWPs in the context of Hong Kong should consider their unique corporate culture and industrial dynamics.

## Figures and Tables

**Table 1:** Sociodemographic characteristics of the caregiver employees (n = 1205).

Variables	Number of CEs (%)
**Female**	665 (55.2)
**Age group**	
*18–19*	11 (0.9)
*20–29*	186 (15.5)
*30–39*	263 (21.8)
*40–49*	330 (27.4)
*50–59*	288 (23.9)
*60–69*	113 (9.4)
*70–79*	13 (1.1)
**Education**	
***Primary school and below***	13 (1.1)
***Secondary school***	70 (5.8)
***High school***	402 (33.4)
***Tertiary education: non-bachelor program***	241 (20.0)
***Tertiary education: bachelor program, master, and above***	479 (39.8)
**Total years of caregiving experience [median (range)]**	10 (0.2–6)
**Average caregiving hours per day**	
*<1*	481 (39.9)
*1–3*	515 (42.7)
*3–5*	126 (10.5)
*>5*	83 (6.9)
**Caregiving dimensions**	
*Physical care*	562 (46.6)
*Emotional care*	245 (20.3)
*Financial support*	973 (80.7)
*Decision-making involvement*	977 (81.1)
**Household income (in HKD)**	
*<25,000*	208 (17.2)
*25,000–29,999*	186 (15.4)
*30,000–39,999*	275 (22.8)
*40,000–49,999*	258 (21.4)
*≥50,000*	276 (22.9)
*Did not disclose*	2 (0.2)
**Industries**	
*Manufacturing*	32 (2.7)
*Construction*	100 (8.3)
*Import/export trade and wholesale*	115 (9.5)
*Retail*	110 (9.1)
*Accommodation and food services*	60 (5.0)
*Transportation, storage, postal, and courier services*	76 (6.3)
*Information and communications*	67 (5.6)
*Financing and insurance*	101 (8.4)
*Real estate*	76 (6.3)
*Professional and business services*	99 (8.2)
*Public administration*	69 (5.7)
*Education*	106 (8.8)
*Human health*	64 (5.3)
*Social work activities, art, entertainment/recreation, and other service activities*	121 (10.0)
*Water supply, sewerage, and waste management/remediation activities*	7 (0.6)
*Agriculture, forestry, and fishing*	2 (0.2)
**Work mode**	
*Full-time employment*	947 (78.6)
*Part-time employment*	234 (19.4)
Both *	24 (2.0)

Notes: * It is common for people in Hong Kong to have both part-time and full-time employment arrangements. According to Census 2021, 2.1% of the youth having full-time employment also have part-time employment concurrently [76].

**Table 2:** Well-being measurements of the caregiver employees.

	Short Warwick–Edinburgh Mental Well-Being Scale	
**Median (IQR)**	24 (21–28)	
**Mean (** **SD)**	24.91 (0.2)	
**By dimensions**	**Variable**	**Mean (SD)**
	Feeling optimistic about the future	3.5 (1.0)
	Feeling useful	3.7 (0.9)
	Feeling relaxed	3.4 (0.9)
	Dealing with problems well	3.6 (0.9)
	Thinking clearly	3.7 (0.9)
	Feeling close to other people	3.6 (0.9)
	Able to make up minds about things	3.7 (0.9)

**Table 3:** Mean SWEMWBS scores by demographic factors.

Demographic Factors	Mean (SD)	Median (IQR)	*p*
**Gender #**			
***Female***	24.9 (5.4)	-	0.7
***Male***	24.9 (5.2)	-	
**Education ^**			
***Primary school and below***	19.6 (6.1)	20 (17–21)	0.001 *
***Secondary school***	23.5 (5.7)	22 (20–27)	
***High school***	24.0 (5.0)	23 (21–28)	
***Tertiary education: non-bachelor program***	24.3 (5.0)	24 (21–28)	
***Tertiary education: bachelor program, master, and above***	26.3 (5.4)	26 (21–30)	
**Average caregiving hours per day ^**			
***<1***	25.5 (5.4)	26 (21–30)	0.001 *
***1–3***	24.6 (5)	24 (21–28)	
***3–5***	24 (5.5)	23.5 (20–28)	
***>5***	24.5 (6.6)	22 (20–30)	
**Household income (in HKD) ^**			
***<25,000***	23.4 (5.2)	21 (21–26)	<0.001 *
***25,000–29,999***	24 (4.6)	21 (20.7–26.0)	
***30,000–39,999***	23.6 (5.1)	22 (21–26)	
***40,000–49,999***	26.3 (5.1)	27 (22–31)	
***≥50,000***	27.4 (5.4)	28 (24–32)	
**Industries ^**			
***Manufacturing***	23.6 (4.3)	23.50 (21–26)	<0.001 *
***Construction***	24.3 (5.4)	22 (21–27)	
***Import/export trade and wholesale***	23.1 (4.7)	21 (20–27)	
***Retail***	23.4 (5.1)	22 (21–26)	
***Accommodation and food services***	22.2 (3.7)	21 (21–25)	
***Transportation, storage, postal, and courier services***	22.3 (4.9)	21 (20–25)	
***Information and communications***	24.6 (4.7)	24 (21–27)	
***Financing and insurance***	25.9 (5.4)	26 (21–30)	
***Real estate***	27.2 (5.3)	27 (24–31.7)	
***Professional and business services***	26.4 (5.9)	26 (21–31)	
***Public administration***	24.6 (5.7)	25 (21–29)	
***Education***	26.7 (5.7)	27 (22–31)	
***Human health***	26.7 (5.4)	27 (21.2–32)	
***Social work activities, art, entertainment/recreation, and other service activities***	26.0 (4.5)	26 (23–29)	
***Water supply, sewerage, and waste management/remediation activities***	27.0 (4.6)	25 (25–32)	
***Agriculture, forestry, and fishing***	23.5 (6.4)	23 (19)	
**Work-mode ^**			
***Full-time employment***	25.1 (5.5)	25 (21–30)	0.019 *
***Part-time employment***	24 (4.6)	23 (21–28)	
***Both***	24.9 (5.4)	25 (21–27.2)	
**Disclosure of CE status ^**			
***Yes***	25.9 (4.9)	26 (21.5–30)	<0.001 *
***No***	24.7 (5.4)	24 (21–28)	
***Inapplicable***	22.2 (6.2)	21 (17.7–26)	
**Care recipients’ utilizing public healthcare services #**			
***Yes***	25.2 (5.3)	-	<0.001 *
***No***	23.3 (5.3)	-	

Notes: # Mann–Whitney U Test; ^ Kruskal–Wallis H test by ranks. * Denotes statistical significance.

**Table 4:** Spearman’s correlation for demographic factors and SWEMWBS scores.

**Demographic Factors**	**Spearman’s Correlation Coefficient**	***p***
**Age of CE**	−0.052	0.07
**Age of care recipients**	0.000	1.0
**Total caregiving experience (years)**	0.088	0.002 *
**CE’s involvement in caregiving dimensions**	−0.017	0.6
**Care recipients’**		
*Social welfare items*	−0.115	<0.001 *
*Number of comorbidities*	0.091	0.002 *
**Care recipient’s support received in caregiving dimensions**		
*Physical care*	0.192	<0.001 *
*Emotional care*	0.282	<0.001 *
*Financial support*	0.185	<0.001 *
*Decision-making involvement*	0.145	<0.001 *

Notes: * Denotes statistical significance.

**Table 5:** Spearman’s correlation between internal and external factors.

Internal and External Factors	Mean (SD)	Median (IQR)	Spearman’s Correlation Coefficient	*p*
**Spillover**				
*Overall*	31.1 (11.7)	32 (20–40)	−0.308	<0.001 *
*Work towards home*	16.2 (6.6)	16 (10–21)	−0.301	<0.001 *
*Home towards work*	14.9 (6)	14 (10–20)	−0.272	<0.001 *
**Self-rate**				
*Total*	151.8 (29.8)	160 (140–170)	0.407	<0.001 *
*Family role*	75.6 (17.1)	80 (70–90)	0.395	<0.001 *
*Work role*	76.2 (16.3)	80 (70–90)	0.330	<0.001 *
**Lubben**				
*Overall*	16.7 (7)	17 (12–21)	0.376	<0.001 *
*Family*	7.5 (3.4)	8 (5–10)	0.388	<0.001 *
*Friends*	9.2 (3.8)	9 (7–12)	0.308	<0.001 *
**Corporate culture**				
*Overall*	11.20 (2.2)	11 (10–13)	0.291	<0.001 *
*Marshall*	8.1 (1.6)	8 (7–9)	0.326	<0.001 *
*LEAD*	3.1 (1.0)	3 (3–4)	0.139	<0.001 *

Notes: * Denotes statistical significance.

**Table 6:** Univariate analysis—Descriptive result of the policy availability and SWEMWBS scores.

Policy Availability	Current Availability n (%)	SWEMWBS Mean (SD)	*p* ^
**Caregiver-inclusive corporate culture**			
*Yes, stated in policy*	127 (10.5)	24.9 (4.5)	<0.001 *
*No, but at discretion*	510 (42.3)	23.2 (4.2)	
*Not at all*	567 (47.1)	26.5 (6.0)	
**Paid caregiver leave (not counting paternity/maternity leave)**			
*Yes, stated in policy*	135 (11.2)	23.6 (4.5)	<0.001 *
*No, but at discretion*	507 (42.1)	23.4 (4.4)	
*Not at all*	562 (46.6)	26.6 (5.9)	
**Unpaid caregivers leave**			
*Yes, stated in policy*	163 (13.5)	23.9 (4.4)	<0.001 *
*No, but at discretion*	481 (39.9)	23.4 (4.4)	
*Not at all*	560 (46.5)	26.5 (5.6)	
**Bereavement leave**			
*Yes, stated in policy*	501 (41.6)	24.3 (4.9)	0.6
*No, but at discretion*	471 (39.1)	24 (4.9)	
*Not at all*	229 (19.0)	26.5 (5.9)	
**Flexible working hours**			
*Yes, stated in policy*	332 (26.7)	24.6 (5.2)	<0.001 *
*No, but at discretion*	486 (40.3)	23.5 (4.7)	
*Not at all*	395 (32.8)	26.2 (5.6)	
**Flexible work locations**			
*Yes, stated in policy*	169 (14.0)	23.4 (4.6)	<0.001 *
*No, but at discretion*	460 (38.2)	24.4 (5)	
*Not at all*	574 (47.6)	26.1 (5.7)	
**Switch to a part-time mode**			
*Yes, stated in policy*	230 (19.1)	23.3 (4.5)	<0.001 *
*No, but at discretion*	473 (39.3)	24.3 (4.9)	
*Not at all*	501 (41.6)	26.1 (5.8)	
**Unpaid leave**			
*Yes, stated in policy*	234 (19.4)	23.30 (4.52)	<0.001 *
*No, but at discretion*	438 (36.3)	24.27 (4.88)	
*Not at all*	531 (44.1)	26.13 (5.78)	
**Aiding medical needs/insurance of employees’ parents**			
*Yes, stated in policy*	308 (25.6)	25.3 (5.1)	<0.001 *
*No, but at discretion*	304 (25.2)	23.5 (4.8)	
*Not at all*	591 (49.0)	25.5 (5.6)	
**Information/Carer Skills/Guide to Community Care Resources**			
*Yes, stated in policy*	61 (5.1)	24.2 (6)	<0.001 *
*No, but at discretion*	445 (36.9)	24.4 (4.4)	
*Not at all*	697 (57.8)	25.9 (5.6)	

Notes: ^ Kruskal–Wallis H test by ranks. * Denotes statistical significance.

**Table 7:** Multivariate analysis—SWEMWBS (Linear Regression analysis).

	Model 1		Model 2	
	Coeff. (95%CI)	*p*	Coeff. (95%CI)	*p*
**Internal Factors**				
**Spillover**				
*Work towards home (M1)*	−0.054 (−0.109–0.000)	0.051	-	-
*Home towards work (M1)*	−0.043 (−0.103–0.018)	0.170	-	-
*Overall (M2)*	-	-	−0.050 (−0.073–−0.027)	<0.001 ***
**Self-rate**				
*Family role (M1)*	0.039 (0.020–0.058)	<0.001 ***	-	-
*Work role (M1)*	0.044 (0.025–0.064)	<0.001 ***	-	-
*Overall (M2)*	-	-	0.041 (0.032–0.050)	<0.001 ***
**External Factors**				
**Lubben**				
*Family (M1)*	0.252 (0.163–0.341)	<0.001 ***	-	-
*Friends (M1)*	−0.006 (−0.095–0.083)	0.900	-	-
*Overall (M2)*	-	-	0.124 (0.082–0.166)	<0.001 ***
**Corporate culture**				
*Marshall (M1)*	0.482 (0.311–0.654)	<0.001 ***	-	-
*LEAD (M1)*	0.108 (−0.160–0.377)	0.428	-	-
*Overall (M2)*	-	-	0.356 (0.240–0.473)	<0.001 ***
**R2**	0.4281		0.4204	
**Adjusted R2**	**0.4038**		**0.3979**	

Notes: *p*-value is denoted as ***: <0.001; Coeff: coefficient, CI: confidence interval. Model 1—dimensional analysis and Model 2—summative analysis with 25 covariates (See Supplementary Table S7).

## Data Availability

Data is unavailable due to privacy or ethical restrictions.

## Supplementary Materials

The following supporting information can be downloaded at: https://www.mdpi.com/article/10.3390/healthcare12101013/s1, File S1: Survey Questions; Figure S1: Histogram of SWEMWBS score distribution; Figure S2: Histogram of the residual of the multivariable regression SWEMWBS (Model 1); Figure S3: Histogram of the residual of the multivariable regression SWEMWBS (Model 3); Table S1: Participant Recruitment based on major industries in Hong Kong (Census 2020 Quarter One); Table S2: Theoretical framework in details; Table S3: Demographic of the first layman interviewed for pilot study; Table S4: List of independent variables; Table S5: Linear Regression for SEWMWBS-Model 1; Table S6: Linear Regression for SEWMWBS-Model 2; Table S7: SWEMWBS Variance Inflation Factor (VIF).
