# Supplementary material for: Caregiver Employees’ Mental Well-Being in Hong Kong
Source: Healthcare (Basel). 2024 May 14;12(10):1013. doi: 10.3390/healthcare12101013 (PMC11121220; doi:10.3390/healthcare12101013)
Supplement: Supplementary file 1 [file healthcare-12-01013-s001.zip › Supplementary Figures.pdf]

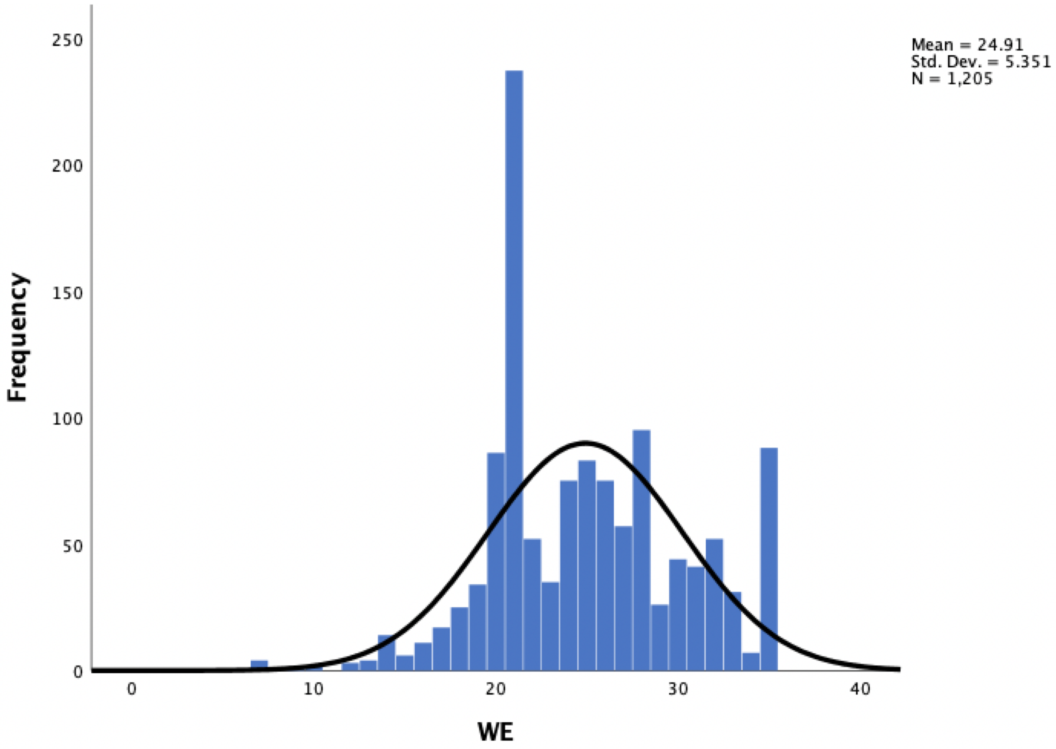

Supplementary Figure S1: Histogram of SWEMWBS score distribution.

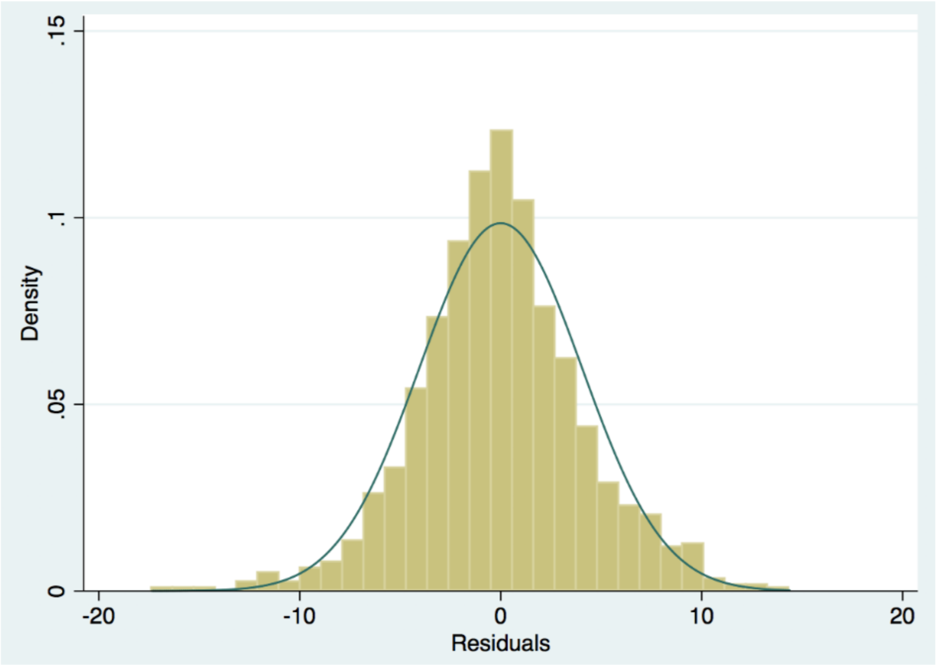

Supplementary Figure S2: Histogram of the residual of the multivariable regression SWEMWBS (Model 1).

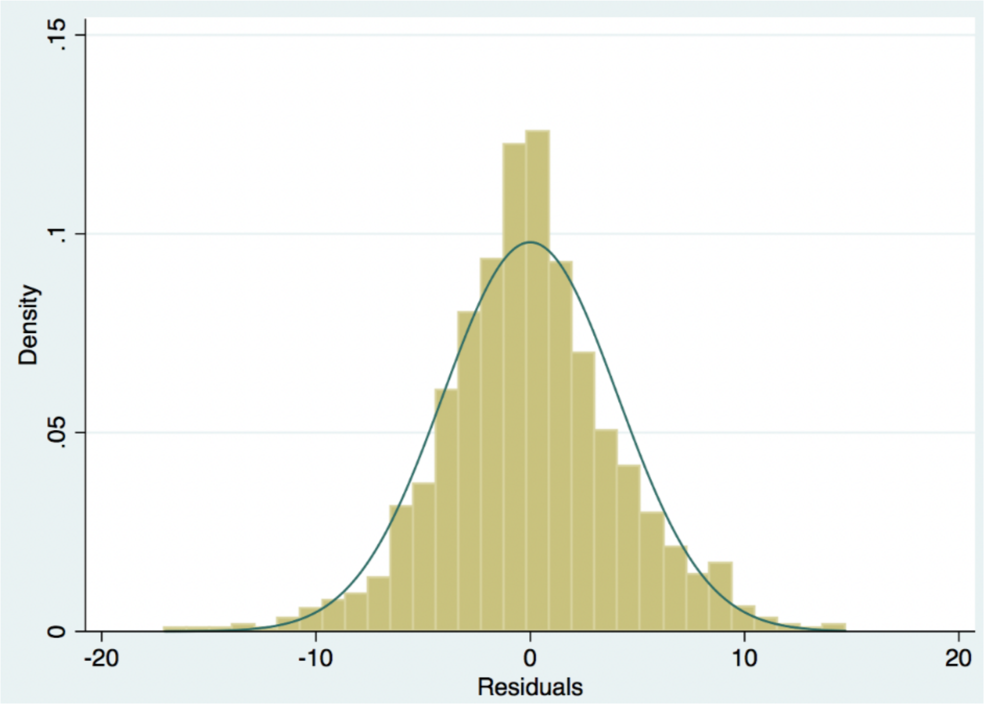

Supplementary Figure S3: Histogram of the residual of the multivariable regression SWEMWBS (Model 3).
