# Supplementary material for: Caregiver Employees’ Mental Well-Being in Hong Kong
Source: Healthcare (Basel). 2024 May 14;12(10):1013. doi: 10.3390/healthcare12101013 (PMC11121220; doi:10.3390/healthcare12101013)
Supplement: Supplementary file 1 [file healthcare-12-01013-s001.zip › Supplementary Material S1 Study Questionnaire.pdf]

Supplementary File S1: Survey Questions

Interview date: ____ start time: _____ end time: ______

Hello Mr/ Ms, I am an interviewer from MOV, and I am entrusted by CUHK JC School of Public Health and Primary Care and Carers HK to carry out a questionnaire survey. Our main goal is to understand the physical and mental state and related determining factors of caregiver employees when taking care of elderly of 65 and/or above and with medical needs, so that we can assist in enhancing policies and legislation in the future. You can be rest assured that the information that you have provided will be kept confidential and will only be used for research and analysis purposes. (Phone Ms. Chan on 2252 8743 during working hours if there are any queries) Thank you for your help!

Part 1-Screening

S1. Are you willing to participate in this study?

1. Willing (or indication of willingness)
2. Not willing (This is the end of the interview, thank you!)

S2. Are you 18 years old or above?

1. Yes
2. No (This is the end of the interview, thank you!)

S3. I will read out the following sentences, please see if they are applicable in describing your current situation.

1. Applicable
2. Not applicable

S3.1 Have provided any care for any family members (it can be any form of care, including money or emotional relief, etc.)

(If S3.1=2, thank the interviewee and end the interview)

S3.2 Of whom you care for age 65 years or older and has medical care needs or chronic illnesses.

(If S3.2=2, thank the interviewee and end the interview)

S3.3 Of whom you care for are your family members, for example parents, grandparents or in-laws, spouse, siblings or children.

(If S3.3=2, thank the interviewee and end the interview)

S3.4 Is in a full-time or part-time job while taking care of this family member.

(If S3.2=2, thank the interviewee and end the interview)

S4. How many months or years have you played the role of "caretaker" in total? (Including all those age 65 or above and has medical care needs or chronic illnesses, including all forms of care, money, or emotional relief etc.)

___ years. If less than a year, follow up with: how many months? ___ months

S5. Are you currently caretaking more than 1 elderly (aged 65 and over and has medical care needs or chronic illnesses)?

1. Yes
2. No

S5a. If "yes", follow up with: How many elderly are you currently caretaking in total?

S6. Following from above, how much time do you spend on each elderly person on average per day? Similarly, I mean all forms of care, which also includes financial or emotional relief etc.

1. Every day, but less than an hour
2. 1-3 hours
3. 3-5 hours
4. 5-7 hours

5. 7-10 hours
6. Over 10 hours
7. Others (please specify): __ hours per week
8. Others (please specify): __ hours per month

S7. At the same time as you are caretaking for elderly people, do you also need to take care of families that are under 65 years old? For example, children, grandchildren, spouse, any disabled family members, etc.? It can be any form of care, money, or emotional relief etc.

1. Yes
2. No

S7a. If "yes", follow up with: How many family members who are below 65 are you caretaking in total?

Part 2- Demographic

The following questions are about the elderly (65+, has medical needs or chronic illnesses) that you spent the most time taking care of. We would like to know more about their needs and support.

A1. Gender of the elderly being cared for:

1. Female
2. Male

A2. Year of birth of the elderly being cared for:

A3. What is your relationship with the elderly?

1. Spouse
2. Father
3. Mother

4. Grandfather
5. Grandmother
6. Siblings
7. Others (please specify): ____

A4. How many children do the elderly currently have (regardless of whether they live together)?

__son(s)

__daughter(s)

A5. What is the medical condition of the person being cared for (multiple choices available)?

1. Hypertension
2. Diabetes
3. Heart disease
4. High cholesterol
5. Cataract
6. Cancer
7. Respiratory diseases
8. Stroke
9. Degenerative arthritis
10. Thyroid disease
11. Gastrointestinal disease
12. Liver disease
13. Parkinson's Disease
14. Kidney disease
15. Dementia or Alzheimer's disease
16. Other, please specify: _____

A6. May I ask who has shared the caretaking role? Please note that this includes all the people who have participated, including yourself, the elderly themselves, the family of the elderly, and even domestic helpers. (Show options) (Multiple choices)

1. Me
2. My spouse
3. Elderly themselves
4. Elderly's spouse
5. My siblings
6. Elderly's (other) children
7. Elderly's son or daughter in law
8. Elderly's grandchildren
9. Other family of the elderly
10. Domestic helper
11. Caretaker nurse
12. Staff at elderly home
13. Others, please specify: _____

	Multiple choices:
1. Which relatives will provide personal care for the elderly? (Note: foreign domestic helpers are included)	
2. Which relatives will provide emotional support for the elderly?	

3. Which relatives will share the expenses of caring for the elderly? (Note: If it is mainly paid by the elderly's CSSA or fruit grant, it will be counted as "elderly self")	
4. Which relatives will discuss major care matters together (the whole hospital, specialist treatment, change of residence, etc.)	

A7. Where is the main residence of the elderly? (Show options) (Single choice)

1. Elderly home
2. Home (original place)
3. Hospital

A8. Which district does this elderly live in? (If required, show options) (Single choice)

1. Hong Kong Island
2. Central and Western District (Kennedy Town, Shek Tong Tsui, Sai Ying Pun, Sheung Wan, Central, Admiralty, Mid-Levels, The Peak)
3. Wan Chai (Wan Chai, Causeway Bay, Happy Valley, Tai Hang, So Kon Po, Jardine's Lookout)
4. Eastern District (Tin Hau, Braemar Hill, North Point, Quarry Bay, Sai Wan Ho, Shau Kei Wan, Chai Wan, Siu Sai Wan)
5. Southern District (Pokfulam, Aberdeen, Ap Lei Chau, Wong Chuk Hang, Shouson Hill, Repulse Bay, Chung Hom Kok, Stanley, Tai Tam, Shek O)
6. Kowloon
7. Yau Tsim Mong District (Tsim Sha Tsui, Yau Ma Tei, West Kowloon Reclamation, King's Park, Mong Kok, Tai Kok Tsui)
8. Sham Shui Po District (Mei Foo, Lai Chi Kok, Cheung Sha Wan, Sham Shui Po, Shek Kip Mei, Yau Yat Estate, Tai Wo Ping, Stonecutters Island)

9. Kowloon City District (Hung Hom, To Kwa Wan, Ma Tau Kok, Ma Tau Wai, Kai Tak, Kowloon City, Ho Man Tin, Kowloon Tong, Beacon Hill)
10. Wong Tai Sin District (San Po Kong, Wong Tai Sin, Tung Tau, Wang Tau Hom, Lok Fu, Diamond Hill, Tsz Wan Shan, Ngau Chi Wan)
11. Kwun Tong District (Ping Shek, Kowloon Bay, Ngau Tau Kok, Jordan Valley, Kwun Tong, Sau Mau Ping, Lam Tin, Yau Tong, Lei Yue Mun)
12. New Territories
13. Kwai Tsing District (Kwai Chung, Tsing Yi)
14. Tsuen Wan District (Tsuen Wan, Lei Muk Shue, Ting Kau, Sham Tseng, Tsing Lung Tau, Ma Wan, Sunny Bay)
15. Tuen Mun District (Tai Lam Chung, So Kwun Wat, Tuen Mun, Lam Tei)
16. Yuen Long District (Hung Shui Kiu, Ha Tsuen, Lau Fau Shan, Tin Shui Wai, Yuen Long, San Tin, Lok Ma Chau, Kam Tin, Shek Kong, Pat Heung)
17. North District (Fanling, Luen Wo Hui, Sheung Shui, Shek Wu Hui, Sha Tau Kok, Luk Keng, Wu Kau Teng)
18. Tai Po District (Tai Po Market, Tai Po, Tai Po Kau, Tai Mei Tuk, Plover Bay, Cheung Mu Tau, Chi Ling Ha)
19. Shatin District (Tai Wai, Sha Tin, Fo Tan, Ma Liu Shui, Wu Kai Sha, Ma On Shan)
20. Sai Kung District (Clear Water Bay, Sai Kung, Tai Mong Tsai, Tseung Kwan O, Hang Hau, Tiu Keng Leng, Ma Yau Tong)
21. Islands District (Cheung Chau, Peng Chau, Lantau Island (including Tung Chung), Lamma Island)

A9. Are you currently living with the elderly? (Show options) (Single choice)

1. Yes
2. No, but it takes within 20 minutes to reach the place where the elderly live.

3. No, it takes 20 minutes to 1 hour to reach the place where the elderly live.
4. No, it takes more than 1 hour to reach the place where the elderly live.

A10. Do the elderly under your care enjoy the following benefits? (Multiple options are available)

1. Age pension
2. Disability allowance
3. CSSA
4. Elderly community care and support services (For example: Elderly District Center / Elderly Support Service Team / Elderly Neighborhood Center / Elderly Activity Center / Carer Support Service / Elderly Holiday Center / Elderly Activity Program / Elderly Day Care Center / Elderly Day Care Service / Improving Home and Community Care services / Integrated Home Care Services / Community Care Services for the Elderly / Elderly Card Scheme)
5. Residential Care Services for the Elderly (Example: residential care nursing / residential care / nursing home places / nursing homes contract homes / residential care homes / elderly emergency residential services / elderly residential respite services)
6. Others, please specify: ______
7. None of the above

The following questions are about you, we would like to know more about your situation

B1. Gender

1. Female
2. Male

B2. Year of birth

B3. What is your marital status

1. Never married
2. Married
3. Widowed
4. Divorce / separation

B4. How many children do you have? This refers to all children regardless of whether they live together.

__son(s)

__daughter(s)

B5. Is your current job:

1. Just a full-time job
2. Only part-time
3. Full-time and part-time

B6. What is your current job industry? (show options) (single choice)

1. Manufacturing
2. Construction industry
3. Import and export trade, wholesale business
4. Retail businesses
5. Accommodation and meal service activities
6. Transportation, warehouse, postal and courier services
7. Information and Communication
8. Real estate activities
9. Professional and business services
10. Public administration
11. Education

12. Human health
13. Social work, community, social and personal service industries
14. Tap water supply, sewage treatment, waste management
15. Agriculture, forestry, fishery, mining, and quarrying
16. 16 Others, please specify: _____

B6a. What is your current position?

1. Management and executive
2. Professionals
3. Paraprofessionals
4. Clerical support staff
5. Service work and sales staff
6. Skilled workers in fishery and agriculture
7. Craftsmen and related personnel
8. Drivers, machines and machine operators/assemblers
9. Unskilled workers
10. Others, please specify: _______

B7. Is your current job (show options) (single option)

1. Just a full-time job
2. Only part-time
3. Full-time and part-time

B8. What is your highest level of education? (if required, show options) (single option)

1. Primary school or below
2. Middle School
3. High School (including sixth form)

4. Post-secondary education- non-degree programs (including diploma or certificate courses)
5. Post-secondary degree- degree programs or above

B9. Please describe your current financial situation

1. No problem at all
2. Can also handle.
3. Not able to cope, quite strenuous
4. Extremely tight, may need to apply for CSSA

B10. What is your total monthly income? Including age pension, caregiver allowance, disability allowance or CSSA (if required, show options) (single option)

1. Less than HK$10,000
2. HK$10,000-HK$19,999
3. HK$20,000-HK$24,999
4. HK$25,000-HK$29,999
5. HK$30,000-HK$39,999
6. HK$40,000-HK$49,999
7. HK$50,000 and above
8. Unwilling to disclose (don’t show)

B11. Have you personally enjoyed the following benefits? (Multiple options can be selected)

1. Age pension
2. Caregiver allowance
3. Disability allowance
4. CSSA – Comprehensive Social Security Assistance Scheme
5. Carer's tax relief

6. Caretaker community care and support services (For example: Elderly District Center / Elderly Support Service Team / Elderly Neighborhood Center / Elderly Activity Center / Carer Support Service / Elderly Holiday Center / Elderly Activity Program / Elderly Day Care Center / Elderly Day Care Service / Improving Home and Community Care services / Integrated Home Care Services / Community Care Services for the Elderly / Elderly Card Scheme)
7. Others, please specify: _____
8. None of the above

Part 3- Well-being indexes

C1. Warwick- Edinburgh Mental: We would like to know more about your well-being. The following are some sentences about feelings and thoughts. Please rate each sentence based on your experience in the past two weeks as follows (single choice) -- 1: never; 2: so rare; 3: sometimes; 4: there are many times (-every other day); 5: keep on (-every day)

- I have always been optimistic about the future.
- I always feel that I am useful.
- I always feel relaxed.
- I have been able to handle the problem properly.
- I can always think clearly.
- I always feel close to people.
- I can always make decisions about things.

C2. Stress and burden. Do you agree or disagree with the following statement: Because of carrying out the caretaking duties, I feel stressful and burdened (Show option 1-2) (single choice)

1. Agree

2. Disagree
3. Don't want to say (don't show)

Part 4 Framework Questions

The following questions are about the needs and supports of the family of the elderly (65+, have medical needs or chronic illnesses)

D1. First, the following questions are about "company support and cultural factors". Do you agree or disagree with the following statements? 1 means strongly disagree; 4 means strongly agree, how much would you give it between 1-4? (show option 1-4) (single choice)

D1a. You blame yourself for not fulfilling your job responsibilities.

D1b. Hong Kong's work culture encourages a clear separation of life and work, i.e. family issues should not be brought to work.

D1c. The corporate culture can help you better manage your role as worker and caregiver.

D2. Do you agree or disagree that the following situation described is a reason that causes anxiety in terms of work? 1 means strongly disagree; 4 means strongly agree, how much would you give it between 1-4? (show option 1-4) (single choice)

D2a The supervisor failed to provide the support you need at work

D3. Then do you agree or disagree that the situation described in the following statements are reasons that you are satisfied at work? 1 means strongly disagree; 4 means strongly agree, how much would you give it between 1-4? (show option 1-4) (single choice)

D3a. Your supervisor cares about the welfare of his subordinates

D3b. You like your immediate boss.

D4 How often does your boss encourage colleagues to work together as a team?

1. Rarely
2. Seldom
3. Sometimes
4. A lot of times
5. Always

D5. The following questions are about "social welfare support". When you are taking care of the elderly (65+, have medical needs or chronic illnesses), have you used any welfare support? For example, the welfare and services from Social Welfare Department, NGOs, charity organizations, Jockey Club charity events etc. (including the interviewee and elderly)

1. Yes
2. No

D6. Is social welfare sufficient to support caregivers? 1 is very inadequate-4 is very sufficient, how much would you give it between 1-4? (show option 1-4 or not applicable) (single choice)

D7. Does your daily work hours hinder your use of social welfare services? 1 means very much, 4 not at all, how much would you give it between 1-4? (show option 1-4 or not applicable) (single choice)

D8. (Ask only if D5=2) What are the reasons why you did not use social welfare support? (show options) (multiple choice)

1. Do not know what social welfare support there are
2. Do not know how to apply
3. Not suitable/ not suitable for us
4. Long waiting time
5. No energy to apply

6. Complex application procedures
7. Relevant services was not provided
8. Hard to afford
9. Worried of going through means test
10. Others (please specify: _________)

D9. Have you ever used home care services/ housekeeping services/ meal services/ home voluntary services? (show options) (multiple choice)

1. Have used government free service
2. Have used government paid service
3. Have used self-paid service
4. Have never used
5. Not willing to disclose (don't show)

D10. Have you ever used home care services? (show options) (multiple choice)

1. Have used government free service
2. Have used government paid service
3. Have used self-paid service
4. Have never used
5. Not willing to disclose (don't show)

D11. Have you ever used allied health services, including physical therapy services, occupational therapy services, speech therapy services? (show options) (multiple choice)

1. Have used government free service
2. Have used government paid service
3. Have used self-paid service
4. Have never used

5. Not willing to disclose (don't show)

D12. Can social welfare support help you to balance the roles of employees and caregivers more effectively? 1 means strongly disagree; 4 means strongly agree, how much would you give it between 1-4? (show options 1-4) (single choice)

D13. The following questions are about "public healthcare support". Have you ever used the following medical services when taking care of the elderly (65+, have medical needs or chronic illnesses)

1. Yes
2. No

D13a. Public healthcare services

D13b. Private healthcare services

D14. Is the medical support information sufficient? 1 is very inadequate, 4 is very sufficient, how much would you give it between 1-4? (show options 1-4 or not applicable) (single choice)

D15. Is medical support information easy to find? 1 is very difficult- 4 is very easy, how much would you give it between 1-4 (show options 1-4 or don't know) (single choice)

D16. (Ask only if D15= 1/2/3) What are the reasons why the information is not easy to find? (Show options) (Multiple choice)

1. The doctor/nurse does not have time to follow up
2. Insufficient information provided by government agencies on illness and care
3. Information provided by government agencies on illness and care is not timely enough
4. Information provided by government agencies on illness and care is inappropriate
5. There is no established sharing platform

6. Does not know how to use the internet
7. Does not know who to go to
8. Others (Note: Please specify ______)

D17. (Ask only if D13a=2) Why did you not use public healthcare service? (Show options) (Multiple choice)

1. Do not know what public healthcare service there are
2. Do not know how to apply
3. Not suitable/ not suitable for us
4. Long waiting time
5. No energy to apply
6. Complex application procedures
7. Relevant services was not provided
8. Hard to afford
9. Worried of going through means test
10. Others (please specify: _________)

D18. (Ask only if D13b=2) Why did you not use private healthcare service? (Show options) (Multiple choice)

1. Do not know what private healthcare service there are
2. Do not know how to apply
3. Not suitable/ not suitable for us
4. Long waiting time
5. No energy to apply
6. Complex application procedures
7. Relevant services was not provided

8. Hard to afford
9. Worried of going through means test
10. Others (please specify: _________)

D19. Do you think that the current holistic medical support services help you balance the roles of employees and caregivers more effectively? 1 means strongly disagree; 4 means strongly agree, how much would you give it between 1-4? (show options 1-4) (single choice)

D20. How much do you agree with the following sentences: 1 means strongly disagree; 4 means strongly agree, how much would you give it between 1-4? (show options 1-4 or don't know) (single choice)

D20a. The information regarding healthcare and social welfare support that you currently know of help you balance the roles of employees and caregivers more effectively

D20a. The healthcare and social welfare services that you currently use help you balance the roles of employees and caregivers more effectively

D21. The following questions are about "public healthcare support". Firstly, regarding family, please base your answers on family members related by blood, marriage, or adoption (they can be your family or relatives).

0=zero; 1=1; 2=two; 3=three or four; 4=five to eight; 5=nine or more

D21a. How many relatives do you meet or talk to at least once a month? (including face to face meeting, phone calls or messages) ___

D21b. How many relatives are able to offer you a safe space to talk about personal issues? ___

D21c. How many relatives make you feel close so you can ask them for help? ___

D22. Regarding friends, please base your answers on friends who live in the same community as you. ("community" can be defined as nearby neighbors, best if same district)

0=zero; 1=1; 2=two; 3=three or four; 4=five to eight; 5=nine or more

D22a. How many friends do you meet or talk to at least once a month? (including face to face meeting, phone calls or messages) ___

D22b. How many friends are able to offer you a safe space to talk about personal issues? ___

D22c. How many friends make you feel close so you can ask them for help? ___

D23. Do you think that family and friends can help you balance the roles of employees and caregivers more effectively? 1 means strongly disagree; 4 means strongly agree, how much would you give it between 1-4? (show options 1-4) (single choice)

D24. The following questions are about the treatment of your role as caregiver at work. Have you ever disclosed your role as caregiver at your company?

1. Yes (Skip to D26)
2. No (Cont. with D25)
3. Not applicable (e.g. one-man company) (Skip to D28)

D25. (Ask only if D24=2) The reasons for not being made public are: (show options) (multiple choices available)

1. Fear of being misunderstood
2. Fear of unfair treatment
3. No need, because other people don't care
4. No need, because I have dealt with it well
5. No need, because I do not require assistance from co-workers
6. Rarely share personal matters with colleagues

7. Others (Note : Please specify ______)

(Skip to D28 after answering D25)

D26. (Ask only if D24=1) What is the reaction of your company and colleagues after your disclosure of your role as caregiver? (show options) (single choice)

1. Negative/ not positive (Cont. with D27)
2. Positive (Skip to D28)
3. Neutral (Skip to D28)

D27. What negative reactions have you experienced? (Show options) (multiple choice)

1. Being discriminated
2. A topic of gossip
3. Being teased
4. Neglected
5. Being viewed as an excuse to skip work
6. Others, please specify: _____

D28. The following questions are regarding your views on managing work and family life. Firstly, we would like to know how do work duties affect family, do you agree or disagree with the following statements? 1 means very strongly disagree, 7 means very strongly agree, how much would you give it between 1-7? (show options 1-7) (single choice)

D28a. My work interferes with your family and family life.

D28b. My work takes up a lot of time and it is difficult to fulfill my family responsibilities.

D28c. Due to work needs, home affairs are not completed.

D28d. Due to my work pressure, it is difficult for me to fulfill my family duties.

D28e. Due to work-related duties, I must make changes to family activities and related itineraries.

D29. Then we would like to know more about how does your family duties affect work, do you agree or disagree with the following statements? 1 means very strongly disagree, 7 means very strongly agree, how much would you give it between 1-7? (show options 1-7) (single choice)

D29a. The demands of my family or spouse / partner will interfere with my work.

D29b. Due to the need to stay at home, I had to put down my work.

D29c. Due to the demands of my family or spouse / partner, I cannot complete the job.

D29d. My family life interferes with my work duties, such as going to work on time, completing daily tasks and working overtime.

D29e. Family-related stress interfered with my ability to perform job duties.

D30. How much do you agree with the following sentences? 1 means strongly disagree; 4 means strongly agree, how much would you give it between 1-4? (show options 1-4) (single choice)

D30a. Going to work can help me relieve the stress as a caregiver (short-term withdrawal from caregiving context)

D30b. Balancing the two roles is easy

D31. If you cannot balance work and caring for the elderly at the same time, would you seek assistance? Please describe the frequency. (Show options 1-5) (Single choice)

1: very rarely/ 2: seldom/ 3: sometimes/ 4: a lot of times/ 5: always

D32. From 0-100, how much would you rate yourself for the following items:

D32a. Do you think you have done your employee's responsibility?

D32b. Do you think you have done your duty as a family member?

Part 5: Policy

E1. Does your job have the following policies? (show option 1-5) (single choice)

please choose:

1. Yes, the company has clearly stated relevant policies
2. No, but management generally handles it as appropriate
3. No relevant support policy at all
4. Not sure
5. The company does not have other support policies (for E1k only)

E1a. Caregiver-inclusive corporate culture

E1b. Paid caregiver leave (not counting maternity leave and maternity leave)

E1c. Unpaid caregiver leave

E1d. Bereavement leave

E1e. Flexible working hours

E1f. Flexible work locations

E1g. Switch to a part-time mode

E1h. Unpaid leave

E1i. Aiding medical needs / insurance of employees' parents

E1j. Information / Carer Skills / Guide to Community Care Resources

E1k. Other policies for supporting caregiver workers, please specify: __________

E2. If the company can provide the following policies, how important do you think the following policies are to support you in taking care of your job and your role as a caregiver? Rate the policies from 1 being least important to 10 being most important (Show option 1-10) (Single choice)

E2a. Caregiver-inclusive corporate culture

E2b. Paid caregiver leave (not counting maternity leave and maternity leave)

E2c. Unpaid caregiver leave

E2d. Bereavement leave

E2e. Flexible working hours

E2f. Flexible work locations

E2g. Switch to a part-time mode

E2h. Unpaid leave

E2i. Aiding medical needs / insurance of employees' parents

Please specify the insured as:

1. *Spouse*
2. *Children*
3. *Parents*
4. *Parent-in-law*
5. *Grandparents*
6. *Siblings*

7. *Others, please specify*

E2j. Information / Carer Skills / Guide to Community Care Resources

E3. Have you thought of other policies that can help you complete the tasks of a caregiver? (Don't show) (can be more than one example)

E4. Overall, do you think that a suitable company policy can help you balance the roles of employees and caregivers more effectively? 1 means strongly disagree; 4 means strongly agree, how much would you give it between 1-4? (show options 1-4) (single choice)

- End of questionnaire -
