# Supplementary material for: Caregiver Employees’ Mental Well-Being in Hong Kong
Source: Healthcare (Basel). 2024 May 14;12(10):1013. doi: 10.3390/healthcare12101013 (PMC11121220; doi:10.3390/healthcare12101013)
Supplement: Supplementary file 1 [file healthcare-12-01013-s001.zip › Supplementary Tables .pdf]

Supplementary Table S1: Participant Recruitment based on major industries in Hong Kong (Census 2020 Quarter One)

**Original Industries**	**Regrouped Industries (for regression analysis)**	**Percentage of the total labor force**	**Sample recruitment**
Manufacturing	Manufacturing	2.7%	31
Construction	Construction	8.3%	93
Import/export trade and wholesale	Import/export trade and wholesale	9.4%	105
Retail<br>Accommodation and food services	Retail, accommodation, and food services	14.2%	158
Transportation, storage, postal/courier services<br>Information/communications	Transportation, storage, postal/courier services, and information/communication	11.9%	131
Financing, insurance<br>Real estate<br>Professional and business services	Financing, insurance, real estate, professional and business services	22.9%	252
Public administration<br>Education<br>Human health<br>Social work activities, art, entertainment and recreation, and other service activities	Public administration, social and personal services	30%	330
Water supply, sewerage, waste management and remediation activities<br>Agriculture, forestry, and fishing	Other industries	0.6%	-
**TOTAL**		100%	1100

Supplementary Table S2: Theoretical framework in details

Concepts	Enablers	Barriers
	External factors	
**Workplace culture**	• Supportive manager (Bernard & Phillips, 2007)<br>• Role empowerment (Lee et al., 2022)	• Apathy (Lee et al., 2022)
**Social Welfare**		• Inadequate social welfare support (lack of crisis intervention and the fragmented and superficial care system) (Lee et al., 2022)
**Healthcare support**		• Difficulties in accessing healthcare support for CEs (Lee et al., 2022)
**Family support**	• Friends and family support (Lee et al., 2022)	
	Internal factors	
**Disclosure of CE role (IVTs)**		• Discrimination & stigmatization (Crenshaw, 1998; Lee et al., 2022),<br>• Fears, self-scrutiny/ judgement (Detert & Edmondson, 2011; Lee et al., 2022; Milliken et al., 2003)
**Spillover between roles (spillover theory)**	• Positive spillover<br>• Role-balancing(Barker, 2014; Lee et al., 2022)	• Negative spillover<br>• Inseparable and non-exclusive role stress and strain (Barker, 2014; Lee et al., 2022)

Supplementary Table S3: Demographic of the first layman interviewed for pilot study

Participant	Age	Gender	Occupation	Relationship with care-recipient	Medical conditions	Caregiving experience
1	30-40	F	Part-time instructor	Father	Cancer	2 years
2	20-30	F	Company Officer	Mother	Cancer	2 years
3	20-30	F	Bank executive	Mother	Cancer	7 years
4	30-40	M	Assistant in education	Mother	Heart diseases, diabetes, stroke	6 years
5	20-30	F	Officer (Financial industry)	Grandparents and mother	Cancer, Alzheimer's, heart diseases, respiratory diseases, mental illness	4.4 years
6	60-70	F	Janitor	Spouse	Diabetes and Stroke	2 years
7	40-50	F	Project Manager	Parents	Heart diseases	3 years
8	60-70	F	Healthcare Assistant	Spouse	Stroke	0.5 years
9	50-60	M	Clerk	Parent	Heart diseases	12 years
10	20-30	F	Business Owner	Parent	Heart diseases	2 years

Supplementary Table S4: List of independent variables

Variable #	Independent variables (Model 1 for sub-scale & Model 2 overall scores)
1	Average caregiving hours per day
2	Household income
3	Industries
4	Work-mode
5	Total caregiving experience (in years)
6	Care-recipients' number of social welfare item
7	Care-recipients' number of comorbidities
8	Disclosure of CE status
9	Care-recipient's' support received in caregiving dimensions: Physical care.
10	Care-recipient's' support received in caregiving dimensions: Emotional care.
11	Care-recipient's' support received in caregiving dimensions: Financial support.
12	Care-recipient's' support received in caregiving dimensions: Decision-making. involvement
13	Care recipient's utilization of public healthcare services.
14-21	Policy availability<br>Questionnaire Items: E1a, E1b, E1c, E1e, E1f, E1g, E1h, and E1i<br>E1j had the multicollinearity problem and was removed from the final regression analyses.
22	Spillover
23	Self-rating
24	Lubben Social Network Scale
25	Corporate culture

Supplementary Table S5: Linear Regression for SEWMWBS - Model 1

	Coefficient	Standard error	t	P>|t|	[95% Conf. Interval]	
Average caregiving hours per day						
2	-0.18588	0.2800395	-0.66	0.507	-0.73532	0.363564
3	-0.10648	0.440262	-0.24	0.809	-0.97028	0.757329
4	0.294184	0.5237849	0.56	0.574	-0.73349	1.321863
Household income						
2	-0.4228	0.4365643	-1.00	0.318	-1.2926	0.420497
3	-0.18427	0.4143626	-0.44	0.657	-0.99726	0.624993
4	0.708927	0.4398667	1.61	0.107	-0.1541	1.571956
5	1.114928	0.4405221	2.53	0.012	0.250614	1.979243
Industries						
2	1.057878	0.8527191	1.24	0.215	-0.61518	2.730933
3	-0.29932	0.840908	-0.36	0.722	-1.9492	1.350565
4	0.904546	0.8136001	1.11	0.266	-0.69176	2.500849
5	0.580062	0.819086	0.71	0.479	-1.027	2.187128
6	1.415707	0.7886681	1.80	0.073	-0.13168	2.963092
7	1.244896	0.7764119	1.60	0.109	-0.27844	2.768234
8	2.253245	1.596365	1.41	0.158	-0.87886	5.385351
Work-mode						
2	-0.17246	0.347256	-0.50	0.620	-0.85378	0.508866
3	0.899312	0.895666	1.00	0.316	-0.85801	2.65663
Care-recipient's utilizing public healthcare services	-0.4191	0.3486975	-1.20	0.230	-1.10325	0.265053
Total caregiving experience (in years)	-0.01658	0.0129165	-1.28	0.199	-0.04193	0.008759
Care-recipient's number of co-morbidities	0.308373	0.1265556	2.44	0.015	0.060068	0.556678
Care-recipient's number of social welfare item	-1.1173	0.2465488	-4.53	<0.001	-1.60104	-0.63357
Care-recipient's' Support received in caregiving dimensions						
a) Physical care	-0.06354	0.1671447	-0.38	0.704	-0.39148	0.2644
b) Emotional care	0.068396	0.1537875	0.44	0.657	-0.23334	0.370131
c) Financial support	-0.11544	0.1944075	-0.59	0.553	-0.49688	0.265988
d) Decision-making	0.476172	0.181679	2.62	0.009	0.119713	0.83263
Disclosure of CE status	-0.47825	0.278161	-1.72	0.086	-1.02401	0.067507
Policy availability E1a						
1	-1.05013	0.4742109	-2.21	0.027	-1.98055	-0.11972
2	0.433695	0.5264565	0.82	0.410	-0.59922	1.466615
E1b						
1	0.072581	0.4544084	0.16	0.873	-0.81898	0.964141
2	0.700034	0.525109	1.33	0.183	-0.33024	1.73031
E1c						
1	-0.60842	0.4312666	-1.41	0.159	-1.45457	0.237738

2	-0.08263	0.4833996	-0.17	0.864	-1.03107	0.865814
E1e						
1	0.163975	0.3511202	0.47	0.641	-0.52493	0.852881
2	-0.15121	0.4179839	-0.36	0.718	-0.97131	0.668881
E1f						
1	-0.94106	0.4531258	-2.08	0.038	-1.83011	-0.05202
2	0.048669	0.4821494	0.10	0.920	-0.89732	0.994658
E1g						
1	0.34158	0.4036218	0.85	0.398	-0.45034	1.133496
2	-0.17314	0.4809053	-0.36	0.719	-1.11669	0.770403
E1h						
1	0.503104	0.4016246	1.25	0.211	-0.28489	1.2911
2	0.503217	0.4713969	1.07	0.286	-0.42167	1.428109
E1i						
1	-0.0752	0.3768125	-0.20	0.842	-0.81451	0.664117
2	0.011029	0.3316312	0.03	0.973	-0.63964	0.661697
Work towards home	-0.05445	0.0278388	-1.96	0.051	-0.10907	0.000169
Home towards work	-0.04259	0.0310021	-1.37	0.170	-0.10342	0.018236
Self-rating - Family	0.039056	0.00976	4.00	<0.001	0.019906	0.058205
Self-rating - Work	0.044317	0.0097845	4.53	<0.001	0.02512	0.063515
Lubben - Family	0.252043	0.0454402	5.55	<0.001	0.162889	0.341198
Lubben - Friends	-0.0057	0.0454326	-0.13	0.900	-0.09484	0.083438
Marshall	0.482149	0.0874145	5.52	<0.001	0.310639	0.653658
LEAD	0.1085	0.1367708	0.79	0.428	-0.15985	0.376847

Supplementary Table S6: Linear Regression for SEWMWBS - Model 2

	Coefficient	Standard error	t	P>|t|	[95% Conf. Interval]	
Average caregiving hours per day						
2	-0.27174	0.2801469	-0.97	0.332	-0.82139	0.277917
3	-0.03867	0.4403027	-0.09	0.930	-0.90256	0.825207
4	0.34607	0.5232166	0.66	0.508	-0.68049	1.37263
Household income						
2	-0.4087	0.4384181	-0.93	0.351	-1.26889	0.451482
3	-0.20478	0.4159393	-0.49	0.623	-1.02086	0.611296
4	0.728061	0.441384	1.65	0.099	-0.13794	1.594064
5	1.120496	0.4420949	2.53	0.011	0.253099	1.987894
Industries						
2	1.251858	0.855297	1.46	0.144	-0.42625	2.929965
3	-0.15216	0.8433439	-0.18	0.857	-1.80682	1.50249
4	0.973865	0.8163878	1.19	0.233	-0.6279	2.575632
5	0.638438	0.8218718	0.78	0.437	-0.97409	2.250964
6	1.576314	0.7907016	1.99	0.046	0.024945	3.127684
7	1.352095	0.7788581	1.74	0.083	-0.17604	2.880227
8	2.481464	1.600448	1.55	0.121	-0.65864	5.621569
Work-mode						
2	-0.07453	0.3469167	-0.21	0.830	-0.75518	0.606128
3	0.849611	0.898394	0.95	0.344	-0.91305	2.612274
Care-recipient's utilizing public healthcare services	-0.31899	0.3481463	-0.92	0.360	-1.00205	0.364083
Total caregiving experience (in years)	-0.01546	0.0129663	-1.19	0.233	-0.0409	0.009976
Care-recipient's number of co-morbidities	0.299469	0.1268834	2.36	0.018	0.050522	0.548416
Care-recipient's number of social welfare item	-1.06746	0.2455536	-4.35	<0.001	-1.54924	-0.58567

Care-recipient's' Support received in caregiving dimensions						
a) Physical care	-0.00617	0.1665551	-0.04	0.970	-0.33295	0.320617
b) Emotional care	0.085669	0.1539935	0.56	0.578	-0.21647	0.387807
c) Financial support	-0.03251	0.193969	-0.17	0.867	-0.41308	0.348058
d) Decision-making	0.449092	0.1817351	2.47	0.014	0.092524	0.805659
Disclosure of CE status	-0.52467	0.2788604	-1.88	0.060	-1.0718	0.022457
Policy availability E1a						
1	-0.8835	0.4741739	-1.86	0.063	-1.81384	0.046834
2	0.594873	0.5268432	1.13	0.259	-0.4388	1.628548
E1b						
1	0.065326	0.4564767	0.14	0.886	-0.83029	0.960941
2	0.820184	0.5266241	1.56	0.120	-0.21306	1.853429
E1c						
1	-0.77541	0.4298834	-1.80	0.072	-1.61885	0.068029
2	-0.24569	0.4818935	-0.50	0.610	-1.19117	0.699792
E1e						
1	0.114445	0.3515635	0.33	0.745	-0.57533	0.804219
2	-0.16461	0.4193468	-0.39	0.695	-0.98737	0.65816
E1f						
1	-0.92968	0.4545112	-2.05	0.041	-1.82144	-0.03792
2	0.121082	0.4836684	0.25	0.802	-0.82788	1.070047
E1g						
1	0.402817	0.4037572	1.00	0.319	-0.38936	1.194996
2	-0.11333	0.4815057	-0.24	0.814	-1.05805	0.831396
E1h						
1	0.399569	0.402441	0.99	0.321	-0.39003	1.189164
2	0.393694	0.4721479	0.83	0.405	-0.53267	1.320055
E1i						
1	0.020325	0.3767607	0.05	0.957	-0.71889	0.759535

2	0.147211	0.3305043	0.45	0.656	-0.50124	0.79566<br>6
Spillover	-0.05042	0.0117566	-4.29	<0.001	-0.07349	-0.02736
Self-rating	0.041223	0.0045974	8.97	<0.001	0.03220<br>3	0.05024<br>3
Lubben	0.124013	0.0213444	5.81	<0.001	0.08213<br>5	0.16589<br>1
Corporate culture	0.356466	0.0596109	5.98	<0.001	0.23950<br>9	0.47342<br>3

Supplementary Table S7: SWEMWBS Variance Inflation Factor (VIF)

Variables		VIF
Average caregiving hours per day		
	2	1.34
	3	1.27
	4	1.21
Household income		
	2	1.75
	3	2.13
	4	2.29
	5	2.41
Industries		
	2	3.89
	3	4.30
	4	5.65
	5	4.94
	6	7.71
	7	8.86
	8	1.33
Work mode		
	2	1.32
	3	1.06
Care-recipient's utilizing public healthcare services		1.17
Total caregiving experience (in years)		1.21
Care-recipient's number of co-morbidities		1.26
Care-recipient's number of social welfare item		1.23
Care-recipient's Support received in caregiving dimensions		
	a) Physical care	1.50
	b) Emotional care	2.37
	c) Financial support	2.35
	d) Decision-making	2.13
Disclosure of CE Status		1.12
Policy availability		
E1a	1	3.94
	2	4.95
E1b		
	1	3.60
	2	4.84
E1c		
	1	3.11
	2	4.04

E1e		
	1	2.08
	2	2.72
E1f		
	1	3.40
	2	4.08
E1g		
	1	2.72
	2	3.93
E1h		
	1	2.62
	2	3.84
E1i		
	1	1.91
	2	2.20
E1j		
	1	6.51
	2	7.00
Spillover		1.33
Self-rating		1.29
Lubben		1.61
Corporate culture		1.22
**Mean VIF**		**2.95**
